# Combination of Durvalumab and Chemotherapy to Potentially Convert Unresectable Stage IV Penile Squamous Cell Carcinoma to Resectable Disease: A Case Report

**DOI:** 10.3390/curroncol30010026

**Published:** 2022-12-26

**Authors:** Hao Xiang Chen, Ching-Chan Lin, Che-Hung Lin, Chi-Rei Yang

**Affiliations:** 1 Department of Urology, China Medical University Hospital, China Medical University, Taichung 40402, Taiwan; 2 Division of Hematology and Oncology, Department of Internal Medicine, China Medical University Hospital, China Medical University, Taichung 404, Taiwan; 3 Division of Hematology and Oncology, Department of Internal Medicine, An Nan Hospital, China Medical University, Tainan 709, Taiwan

**Keywords:** penile cancer, squamous cell carcinoma, durvalumab, immunotherapy, combination

## Abstract

Penile squamous cell carcinoma is a rare disease entity with poor overall survival in an advanced stage. Few studies have investigated the role of immunotherapy in advanced penile squamous cell carcinoma. Herein, we report a case of stage IV unresectable penile squamous cell carcinoma presenting with anal bleeding and urethra obstruction who responded dramatically to combination therapy of durvalumab and cisplatin-based chemotherapy. The patient had HPV-positive penile squamous cell carcinoma, cT3N3M0, with concomitant anus squamous cell carcinoma. After 2 months of the combination treatment, almost all bulky inguinal lymph nodes shrank, and the main tumor of the anus and penis responded completely. A durable response was seen 16 months after initiating the combination therapy. This case report highlights the potential role of the combination of immunotherapy and chemotherapy in patients with advanced penile cancer. The promising results of this combination resulted in the conversion of unresectable disease to a potentially curable disease.

## 1. Introduction

Penile squamous cell carcinoma (PSCC) is a rare malignant disease in men with a reported prevalence ranging from around 1 in 100,000 males in industrialized countries such as the USA and Europe [1,2] to 1 to 2% of malignant disease in certain African countries [3,4,5]. It is especially prevalent in areas with a high prevalence of human papillomavirus (HPV) infection [6].

Delayed diagnosis is a serious problem in PSCC, and around 65% of PSCC patients have a delay in diagnosis of more than 6 months. The most common cause of delayed diagnosis is due to embarrassment over the location of the symptoms [7]. Delayed diagnosis of PSCC will ultimately result in a more advanced stage of the disease [8], and the prognosis for advanced disease is poor, especially for those with extranodal involvement. The 5-year survival rate for PSCC with the extranodal disease is 0 to 71% based on data from different study periods [9,10]. 

Currently, the first-line systemic therapy for stage IV PSCC is cisplatin-based chemotherapy (paclitaxel, ifosfamide, and cisplatin or 5-fluorouracil(5-FU) and cisplatin) [11,12]. The average response rate to cisplatin-containing regimens is 26%, and the median survival is 5.5 months [13]. Immunotherapy has changed the therapeutic landscape of many different cancers, and the combination therapy of PD-1/PD-L1 blockade with other immune checkpoint inhibitors (ICIs) or conventional therapy is not new to genitourinary cancers [14]. However, due to its rarity, no randomized clinical trial has yet been conducted on immunotherapy for advanced penile cancer. 

Herein, we report a case of metastatic penile cancer with concomitant anal SCC who showed a dramatic response to the combination of durvalumab and chemotherapy (cisplatin and 5-FU).

## 2. Case Presentation

**Figure 1:**
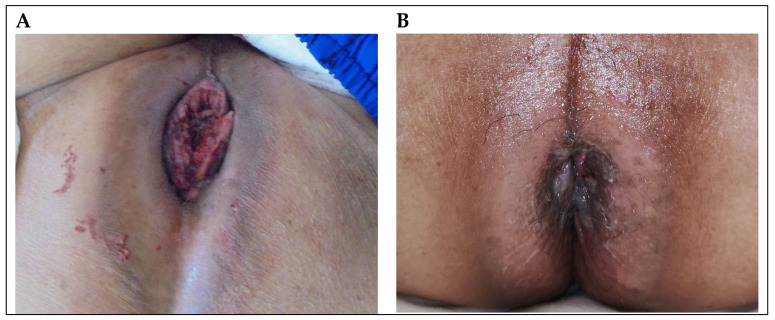
(**A**) Gross appearance of anal SCC protruding from the anus. (**B**) The tumor was almost not visible externally 2 months after combination therapy.

**Figure 2:**
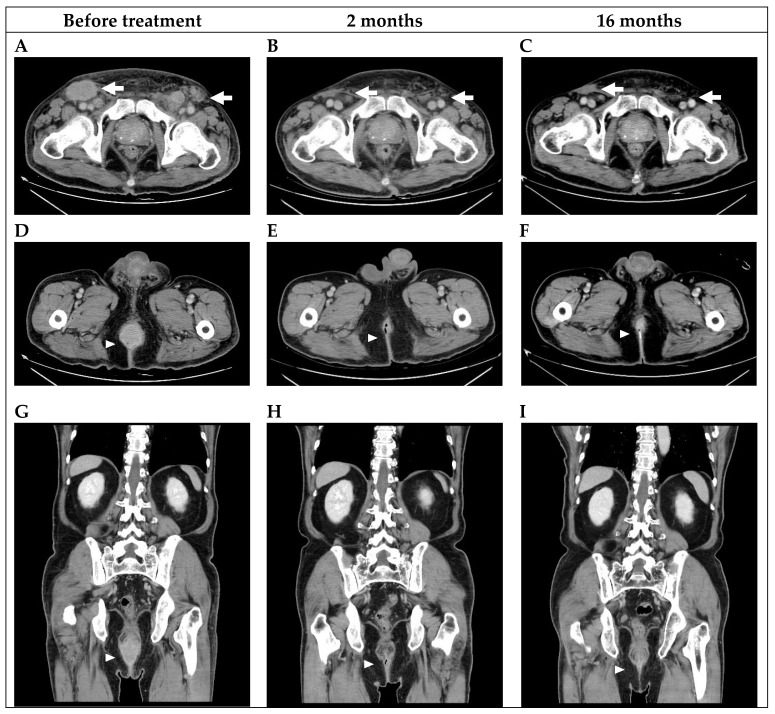
(**A**–**C**) Bilateral inguinal lymphadenopathy. Arrows: The bulky bilateral inguinal lymph nodes shrank 2 months after the combination treatment of durvalumab and chemotherapy. The effect persisted at 16 months. (**D**–**F**) Axial view of the rectal mass. (**G**–**I**) Coronal view of the rectal mass. Arrowhead: The rectal mass protruding from the anus shrank 2 months after combination treatment, and a complete response was noted at 16 months.

The patient was a 64-year-old man with syphilis and human immunodeficiency virus infection which had been well-controlled with an undetectable viral load for 20 years. He was diagnosed with PSCC in August 2018. He initially presented several well-defined erythematous patches with erosion on the dorsal glans and coronal sulcus. The lesion was not painful and had no discharge. A biopsy of the lesion showed deep invasive keratinizing squamous cell carcinoma, grade 3, with overlying carcinoma in situ. HPV 16/18/31/33/51 was detected in the specimen. Abdominal computed tomography (CT) and chest CT showed lymphadenopathy of bilateral inguinal lymph nodes. He refused penectomy because he was concerned about the loss of sexual function and did not return for continued care until he suffered from difficult voiding, pain and bleeding 3 years later after his first evaluation. A physical examination showed that the tumor involved the anus, perianal region, penis and perinium (Figure 1A). In addition, multiple fixed, bilateral palpable inguinal lymph nodes were also noted. CT revealed that the penile tumor obstructed the urethra, and a 3.8 × 3.5 cm protruding mass from the anus was noted with multiple enlarged lymph nodes over paraaortic, paracaval, perirectal, bilateral parailiac and bilateral inguinal regions (Figure 2A,D,G). A biopsy of the anal lesion showed squamous cell carcinoma, and immunohistochemical staining results for P16 and P40 were positive. The stage according to TNM staging of PSCC was cT3N3M1. The PD-L1 expression of the lesion was tumor proportion score(TPS) category of less than 1% and combined positive score (CPS) category between 1 and 10.

The patient received systemic therapy with durvalumab 240 mg, cisplatin 100 mg, and 5-fluorouracil 2000 mg every 2 weeks for six cycles from March 2021 to June 2021. The treatment course was smooth without significant adverse events. After six cycles of durvalumab and chemotherapy combination therapy, CT showed that the bilateral inguinal lymph nodes and anal protruding mass had shrunk dramatically (Figure 2B,E,H). Externally, no visible tumor mass could be seen in either the genital area or perianal region (Figure 1B). A physical examination at this time showed only one palpable left inguinal lymph node. 

The patient then discontinued durvalumab due to financial concerns. As the disease had become potentially resectable a staged operation was planned; however, he refused further surgery and was treated with chemotherapy alone after discontinuation of durvalumab. Five months later, CT showed no anal tumor and regression of bilateral lymph nodes. The patient was then treated with chemotherapy with six cycles of cisplatin and 5-FU from November 2021 to January 2022, and subsequently switched to uracil-tegafur since July 2022. At 16 months, CT showed stable disease over bilateral inguinal lymph nodes, anal tumor and retroperitoneal lymph nodes (Figure 2C,F,I). He declined further consolidation surgery with the hope of preserving the penis, so he received radiotherapy as consolidation therapy.

## 3. Discussion

We present a case of bulky metastatic PSCC with concomitant locally advanced anal squamous cell carcinoma who responded dramatically to the combination of durvalumab and chemotherapy. This case demonstrates that the combination of immunotherapy and chemotherapy can potentially convert unresectable PSCC to potentially resectable disease. To the best of our knowledge, this is the first case report of the combination of durvalumab and conventional chemotherapy in a patient with metastatic PSCC. The limitations of this case report are that the patient declined consolidation surgery, and that durvalumab was discontinued after six cycles due to financial concerns.

Advances in the treatment of PSCC have been relatively slow compared with other urological cancers. Current standard treatment for unresectable/metastatic PSCC is chemotherapy with cisplatin-containing regimens (TIP or 5-fluorouracil+cisplatin). However, the response rates to these regimens are disappointing, and the average response rate to cisplatin-containing regimens for PSCC is only 26% [13]. This corresponds to the poor overall survival of metastatic PSCC, with a 5-year overall survival rate of 0% [9,13].

Immunotherapy has changed the treatment algorithm of different kinds of cancers, including urological cancers such as urothelial carcinoma and renal cell carcinoma, and non-urological cancers such as squamous cell carcinoma of the head and neck and lung cancer. However, as PSCC is a rare malignant disease, no randomized control trials have yet been designed specifically to investigate the efficacy of immunotherapy for PSCC. Several case reports have presented cases of advanced PSCC which responded remarkably to immunotherapy (Table 1). Trafalis et al. presented a case of recurrent metastatic PSCC with a partial response to nivolumab after failure of chemoradiation, with a time to response to nivolumab of 2.5 months [15]. In addition, Chahoud et al. demonstrated an extremely durable response of chemoradiation-refractory metastatic PSCC to pembrolizumab in patients with high tumor mutational burden and PD-L1-expressing penile cancer [16]. Another case report demonstrated a complete response of T3N3M0 PSCC to neoadjuvant tislelizumab combination chemotherapy [17].

Many studies have shown the efficacy of the combination of chemotherapy and immunotherapy in cancer treatment. For example, a multicenter randomized phase 3 trial reported that the combination of toripalimab (anti-PD1) with platinum-based chemotherapy resulted in a median progression-free survival of 11.7 months versus 8.0 months in the placebo arm, with a hazard ratio of 0.52 for metastatic or recurrent head and neck cancer [18]. The CAPTAIN-1st trial demonstrated an objective response rate of 87% in patients receiving camrelizumab plus platinum-based chemotherapy. Moreover, the medial progression-free survival of the combination therapy group was significantly longer than that of the placebo group (9.7 months versus 6.9 months) [19]. Data regarding combination therapy for cervical cancer is also available. The KEYNOTE-826 trial showed that the addition of pembrolizumab to platinum-based chemotherapy significantly prolonged overall survival and progression-free survival in patients with recurrent, persistent or metastatic cervical cancer [20]. 

Previous studies have shown that around 40–60% of metastatic PSCC has a high PD-L1 expression [21,22], and PD-L1-expressing solid tumors have been associated with a more robust response to immunotherapy [23]. PD-L1-positive melanoma and non-small cell lung has also been reported to have a significantly higher response rate compared to PD-L1-negative disease [24,25]. However, the association of PD-L1 expression and the response to immunotherapy in PSCC is unclear. A few studies have reported that PD-L1-positive PSCC is correlated with a poor prognosis. For example, Bacco et al. reported that PD-L1 expression was associated with lymph node metastasis and worse clinical outcomes [21,26]. Tumor mutational burden (TMB) has been suggested as a potential predictive biomarker for immunotherapy. TMB varies among different cancer and the TMB cutpoint used in clinical trials varies widely [27]. The decision of FDA to approve pembrolizumab for solid tumor with TMD > 10 mutations/megabase was based on the results of KEYNOTE-158 which the objective responses rate was 29% and more than half of the responses were greater than 2 years [28].

In a phase II basket trial of pembrolizumab for advanced PSCC, one patient with T3 PSCC with microsatellite instability-high (MSI-H) showed a durable response to pembrolizumab after failure of platinum-based chemotherapy. Moreover, the patient remained disease free 38.7 months after pembrolizumab and consolidation surgery [29]. Another phase II study (NCT03333616) of nivolumab plus ipilimumab for advanced genitourinary cancer included 6 penile cancer cases [30]. In that study, only 2 of the patients achieved a partial response while the others had progression of the disease. In a phase 2 clinical trial of atezolizumab with or without radiation to stage IV penile cancer, the objective response rate was 30%(10% complete response and 20% partial response) [31]. This study showed that a subset of penile cancer patients may respond well to immunotherapy. Currently, the NCCN guidelines include pembrolizumab as second-line treatment for unresectable/metastatic PSCC with MSI-H or mismatch repair deficient [22] or tumor mutational burden-high (TMB-H, >10 mutations/megabase) solid tumors [32]. 

Consolidation surgery is recommended if the initially unresectable PSCC responds to systemic therapy. In a case series of 10 patients with advanced PSCC receiving chemotherapy who underwent consolidation surgery, 4 remained disease-free at a median follow-up time of 62 months [33]. Our case shows that the combination of immunotherapy and chemotherapy has the potential to convert initially unresectable disease to resectable status. Consolidation surgery may be beneficial for these patients.

There are several ongoing clinical trials of immunotherapy for PSCC that are expected to be completed in 2023. For example, the NCT04224740 phase II trial was designed to investigate the combination of pembrolizumab with standard of care chemotherapy in the first-line setting of stage IV PSCC or recurrent locally advanced disease. In addition, NCT03391479 is a study of avelumab treatment for patients with PSCC who are ineligible or have disease progression after platinum-based chemotherapy, and it is expected to be completed in June 2023. NCT03686332 is another phase II study investigating atezolizumab with or without radiotherapy for unresectable PSCC, and it is expected to be completed in December 2023. These clinical trials may be able to provide evidence for the use of immunotherapy in patients with advanced penile cancer.

The limitation of our case is we do not have the tumor mutation burden and MSI data for the patient. Our case showed that immunotherapy might be beneficial in patients with low PD-L1 expression. In adition, more cases will be needed to prove the concept of combination therapy of immunotherapy and chemotherapy in advanced PSCC.

## 4. Conclusions

Our case demonstrates that the combination of durvalumab and chemotherapy can be very effective in a systemic therapy setting for unresectable PSCC, and that it has the potential to convert unresectable PSCC to resectable and curable disease. Further clinical trials of the combination of immunotherapy and chemotherapy are warranted.

## Figures and Tables

**Table 1:** Case reports of immunotherapy on advanced penile cancer.

Study	Age	Staging	Treatment	Prior Treatment	Response	Duration of f/u	HPV	PD-L1	MSI	TMB
Trafalis [15]	47	N/A	Nivolumab	Chemoradiation	PR	9 months	Negative	Positive	Negative	High
Chahoud-1 [16]	64	TxN3M0	Pembrolizumab	C/T+PLND+RT	CR	38 months	Unknown	N/A	Ambiguous	High
Chahoud-2 [16]	85	TxpN3M0	Pembrolizumab	Partial penectomy, chemotherapy, radiation	PR	18 months	Negative	Positive	Negative	Low
Dou [17]	32	T3N3M0	Tislelizumab+C/T	Nil	CR	8 months	N/A	Positive	N/A	N/A

AE: Adverse events, HPV: Human papillomavirus, PD-L1: programmed death ligand-1, MSI: microsatellite instability, TMB: tumor mutation burden, C/T: chemotherapy, PLND: pelvic lymph node dissection, RT: radiotherapy, N/A: not available.

## Data Availability

All of the data are available upon request to the corresponding author.
